# Transcriptomic alterations in roots of two contrasting *Coffea arabica* cultivars after hexanoic acid priming

**DOI:** 10.3389/fgene.2022.925811

**Published:** 2022-10-28

**Authors:** Ilara G. F. Budzinski, Paula O. Camargo, Samara M. C. Lemos, Romain Guyot, Natália F. Calzado, Suzana T. Ivamoto-Suzuki, Douglas S. Domingues

**Affiliations:** ^1^ Group of Genomics and Transcriptomes in Plants, Department of Biodiversity, Institute of Biosciences, São Paulo State University, UNESP, Rio Claro, Brazil; ^2^ Graduate Program in Biological Sciences (Genetics), Institute of Biosciences, São Paulo State University, UNESP, Botucatu, Brazil; ^3^ Institut de Recherche pour le Développement (IRD), Université Montpellier, Montpellier, France; ^4^ Department of Genetics, “Luiz de Queiroz” College of Agriculture, University of São Paulo, ESALQ/USP, Piracicaba, Brazil

**Keywords:** RNA-seq, coffee, hexanoic acid, priming agent, elicitation, root

## Introduction

Plants have the capacity to enter a state of alert that enables them to respond rapidly and robustly after exposure to stress (Aranega-Bou et al., 2014). This phenomenon is known as priming and can be described as an induced state whereby plants are pre-exposed to an inducing agent (elicitor), thus improving their perception and/or amplification of defense response-inducing signals (Aranega-Bou et al., 2014; Tugizimana et al., 2018). Hexanoic acid (Hx), a monocarboxylic acid, is a natural priming agent with proven efficiency in a wide range of host plants and pathogens (Llorens et al., 2016), including coffee pathogens. Coffee (*Coffea* spp.) is one of the most important agricultural commodities in the world. Brazil is the largest producer and exporter of *Coffea arabica* L. (Brazilian Coffee Exporters Council, 2021). The genus *Coffea* comprises 124 species (Davis et al., 2011). The most planted one is *C. arabica*, the only allotetraploid species in the genus. As many other plants, *Coffea* spp. are sensitive to a diverse range of biotic and abiotic stress. It is known that priming leads to changes at the transcriptional, physiological, metabolic and epigenetic levels (Baccelli et al., 2020). A transcriptional reprogramming may occur after priming stimulation, affecting a huge number of genes (Cervantes-Gámez et al., 2016; Baccelli et al., 2020). Within this context, our aim was to investigate the effect *per se* of Hx application. We hypothesize if Hx application could modulate genes related to defense response, in *C. arabica*, being a potential eliciting agent to this crop. To test this, Hx was applied in the roots of two Brazilian *C. arabica* cultivars: Catuaí Vermelho and Obatã. Cultivars were chosen based on their distinct breeding histories and contrasting resistance to rust, the major disease in Arabica coffee worldwide (Talhinhas et al., 2017). Catuaí Vermelho is susceptible to rust, and is one of the most planted cultivars in Brazil, while Obatã is described as a moderately resistant cultivar (Del Grossi et al., 2013). In the present work, transcriptomic analysis of roots were performed, revealing different molecular responses. Based on FPKM ratio and statistical analyses, 1,545 differentially expressed genes (DEGs) were found. Functional annotation of DEGs through Blast2GO showed that primary, organic substance and cellular metabolic processes were mainly affected by priming, in both cultivars. Here, we present an RNA-seq dataset containing raw files and an initial exploration of differentially expressed genes in two *C. arabica* cultivars. Besides, these data could contribute to the identification of key genes differentially expressed in response to Hx.

## Material and methods

### Plant material

Plant material and experimental setup used in this work was the same described in a previous publication from our group (Budzinski et al., 2021).

Two commercial cultivars of *C. arabica* (five-month-old plants) were used, Catuaí Vermelho IAC 144 and Obatã IAC 1669-20. Both cultivars are inbred lines of *C. arabica* (Maluf et al., 2005); however, Catuaí is derived from a cross between Catuaí Amarelo 476 × Mundo Novo 374-19, while Obatã is derived from interspecific crosses between (Villa Sarchi × Hybrid of Timor) × Catuaí Vermelho; clarifying that Villa Sarchi is a *C. arabica* cultivar and Hybrid of Timor is a natural *C. arabica* x *C. canephora* hybrid (Lashermes et al., 2000; Maluf et al., 2005). These cultivars were chosen due to their contrasting response to rust, with Obatã being the resistant one (Maluf et al., 2005; Krohling et al., 2018). Plants were selected based on size uniformity and were transferred to pots containing 3 L of aerated nutrient solution (ANS), adapted from Clark, 1975) by de Carvalho et al. (2013). The experiment was carried out as described in Silva et al. (2020), under controlled temperature (23 ± 2°C) and light/dark cycle (12h/12h, photosynthetically active photon flux density of ∼400 μmol m^−2^.s^−1^). The following treatments were applied: (a) ANS (control); (b) ANS + hexanoic acid (Merck, final concentration 0.55 mM) for 48 h. Three plants per pot were grown into six plastic pots in which three pots received each treatment. The experiments were repeated 3 times to obtain biological replicates. The potted plants were grouped in “pools” (made of 9–18 plants), which were considered a biological replicate. Three biological replicates were used. We collected plant secondary roots within the 3rd hour of the light period and stored at -80°C for further analyses.

### Total RNA extraction and quality control

All steps from total RNA extraction until gene expression analysis were the same as described in Budzinski et al. (2021).

Total RNA from root pools were isolated using the RNeasy Plant kit (Qiagen, Hilden, North Rhine-Westphalia, Germany). Total RNA samples were purified using the RNeasy Minielute Cleanup kit (Qiagen, Hilden, North Rhine-Westphalia, Germany). The purity of RNA was determined using a NanoDrop ND-100 spectrophotometer (Thermo Scientific, San Jose, CA, United States). RNA concentrations were measured by a Qubit fluorometer (Thermo Fisher Scientific, Wilmington, DE, United States).

### Library preparation, and RNA-seq

Poly(A) RNA sequencing library was prepared following Illumina’s TruSeq-stranded-mRNA sample preparation protocol (Illumina Technologies, SanDiego, CA). Paired-end sequencing (2 X 150 bp) was performed on Illumina’s NovaSeq 6000 sequencing system at LC Sciences (Houston, TX, United States). Data was deposited into the European Nucleotide Archive (ENA), submission PRJEB52366.

### RNAseq analysis and gene expression analysis

All steps mentioned here are the same as described in Budzinski et al. (2021). Adaptor contamination, low quality bases and undetermined bases were removed by using Cutadapt (Martin, 2011) and in house PERL scripts. Sequence quality was verified using FastQC (Andrews, 2010). HISAT2 (Kim et al., 2015) was used to map reads to the *Coffea arabica* genome (ftp://ftp.ncbi.nlm.nih.gov/genomes/all/GCF/003/713/225/GCF_003713225.1_Cara_1.0/).

StringTie (Pertea et al., 2015) was used to assemble the mapped reads and to detect the expression level for mRNAs by calculating FPKM. The differentially expressed genes (DEGs) were selected with log2 (fold change) >1 or log2 (fold change) <-1 and with statistical significance (*p* value <0.05) by R package edgeR (Robinson et al., 2010). A second analysis was done on the differentially expressed mRNAs and only the ones with FPKM (ratio) ≥ 2 or FPKM (ratio) ≤ -2; coefficient of variation ≤30% and average FPKM ≥5 were selected for further analyses. Genes found specifically in one condition (control or plants exposed to Hx) were also described as DEGs.

Sequence annotation and gene ontology (GO) enrichment analysis of DEGs were performed using *Blast2GO* (Conesa et al., 2005), at the *BioBam* (Götz et al., 2008) platform. Sequences were annotated by blasting nucleotide sequences against the NCBI NR database (BLASTX, evalue ≤1.10^−5^). The hypergeometric distribution was used to test whether the GO function set was significantly enriched (*p* < 0.05). Pathway mapping was done using MapMan software (Thimm et al., 2004) with the *Arabidopsis thaliana* mapping file (http://mapman.gabipd.org/). TAIR IDs were retrieved from NCBI (https://www.ncbi.nlm.nih.gov).

### Overall data annotation, differentially expressed genes and gene ontology analysis

Quality control and mapping information are available in Table 1. About 67.12 Gb total clean bases were obtained by RNA-seq after quality check, with an average of 5.6 Gb for each sample. The lowest value of Q30 (percentage of bases with sequencing error rate lower than 1∘) was 97.36%. The GC content ranged from 45 to 52%.

**TABLE 1:** Summary of sequencing data quality

Sample	Raw data	Valid data	Valid data gb (G)	Valid ratio (reads)	Q30%	GC content%
CC_root1	53,455,648	38,232,894	5.73	71.52	99.03	46
CC_root2	52,404,378	38,309,030	5.75	73.1	99.02	45.5
CC_root3	51,672,842	36,455,566	5.47	70.55	98.99	45
OC_root1	42,011,570	37,025,650	5.55	88.13	97.6	45
OC_root2	42,381,098	37,588,930	5.64	88.69	97.36	45
OC_root3	41,533,684	36,290,882	5.44	87.38	97.36	45.5
CHX_root1	44,422,470	32,902,840	4.94	74.07	97.41	51
CHX_root2	51,270,376	50,002,294	7.5	97.53	97.84	51
CHX_root3	46,166,432	40,680,326	6.1	88.12	98.59	52
OHX_root1	33,581,294	32,733,990	4.91	97.48	98.12	51
OHX_root2	41,411,214	35,239,206	5.29	85.1	98.57	51
OHX_root3	32,963,354	31,991,596	4.8	97.05	98.18	52

**FIGURE 1:**
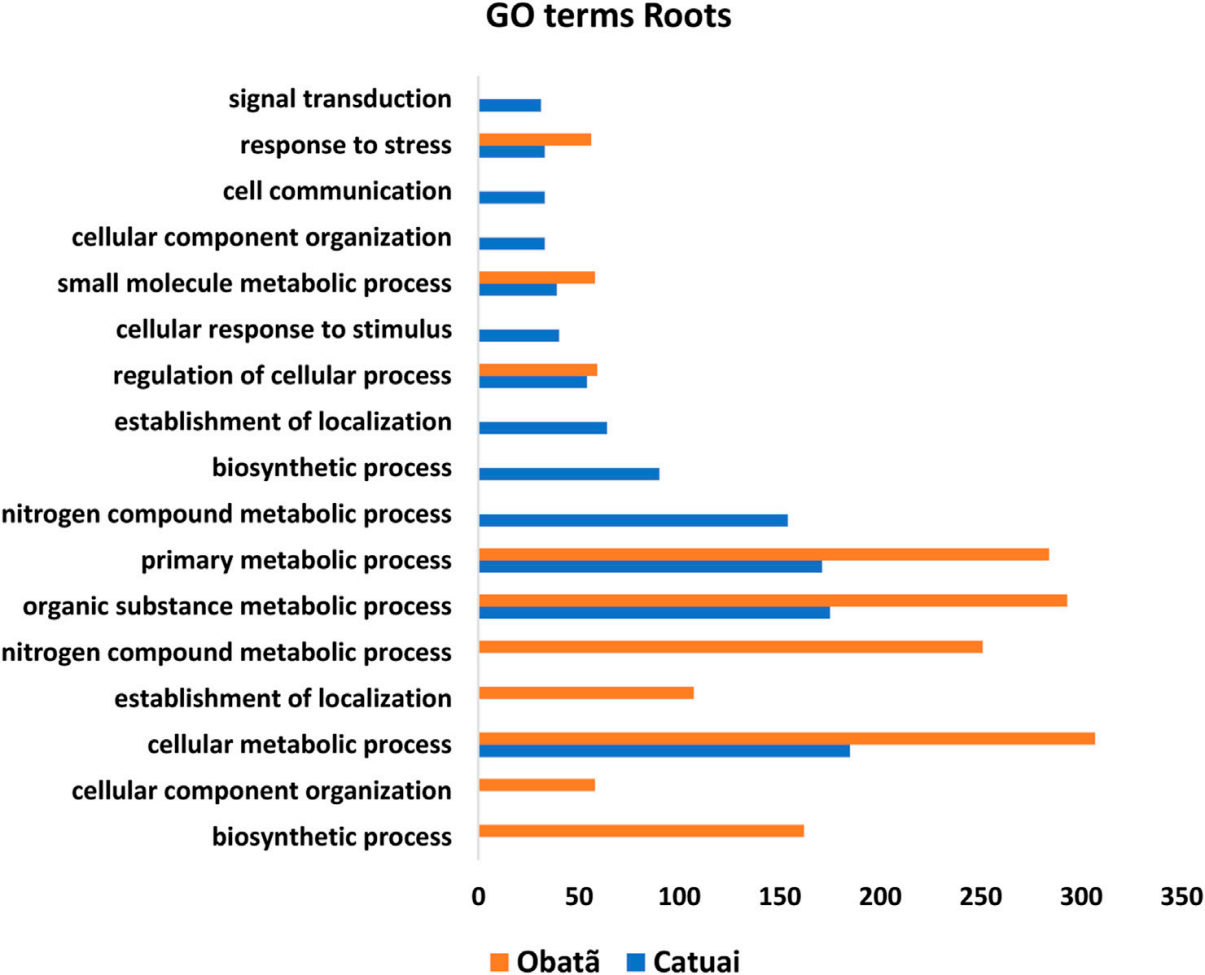
Gene ontology (GO) functional enrichment analysis of DEGs from C. *arabica* Catuaí and Obatã cultivars.

As a preliminary analysis to identify genes and functional categories potentially modulated by Hx application, the first step of our work was to identify the DEGs based on FPKM and statistical analysis. Based on FPKM ratio and statistical analyses, 1,545 DEGs were found in total, 557 and 988 in Catuaí and Obatã, respectively (Supplementary Table S1). From these, 157 DEGs were found in both cultivars, while 400 and 831 DEGs were specifically found in Catuaí and Obatã cultivars, respectively (Supplementary Tables S2, S3). We hypothesize that the discrepancy between the number of specific DGEs, found in each cultivar, is related to differences in rust resistance, reinforcing that molecular mechanisms of defense are differentially recruited depending on cultivar tolerance. Most of the DEGs have a role in plant defense, indicating the modulation of this mechanism in roots by priming. Blast2GO analysis showed that primary, organic substance and cellular metabolic processes were mainly affected by priming, followed by response to stress, small molecule metabolic process and regulation of cellular process (Figure 1, Supplementary Table S5). Pathway analysis of DEGs using MapMan showed differences in the activity of cellular metabolisms due to Hx (Supplementary Table S3). The dataset presented here indicates that hexanoic acid modulates plant defense mechanisms in *C. arabica*. Moreover, we are providing useful data for further investigations on *C. arabica* root responses to Hx.

## Data Availability

The datasets presented in this study can be found in online repositories. The names of the repository/repositories and accession number(s) can be found below: https://www.ebi.ac.uk/ena, PRJEB52366. All supplementary files are available on https://doi.org/10.5281/zenodo.6467813.
